# 3D Morphology of Different Crystal Forms in β-Nucleated and Fiber-Sheared Polypropylene: α-Teardrops, α-Teeth, and β-Fans

**DOI:** 10.1021/acs.macromol.3c00788

**Published:** 2023-07-07

**Authors:** Shu-Gui Yang, Liang-Qing Zhang, Changlong Chen, Jiaming Cui, Xiang-bing Zeng, Liying Liu, Feng Liu, Goran Ungar

**Affiliations:** † Shaanxi International Research Center for Soft Matter, State Key Laboratory for Mechanical Behaviour of Materials, Xi’an Jiaotong University, Xi’an 710049, China; ‡ College of Material Science and Engineering, Xi’an University of Science and Technology, Xi’an 710054, China; § Department of Materials Science and Engineering, University of Sheffield, Sheffield S1 3JD, U.K.; ∥ Biomedical Experimental Center of Xi’an Jiaotong University Health Science Center, Xi’an 710116, China

## Abstract

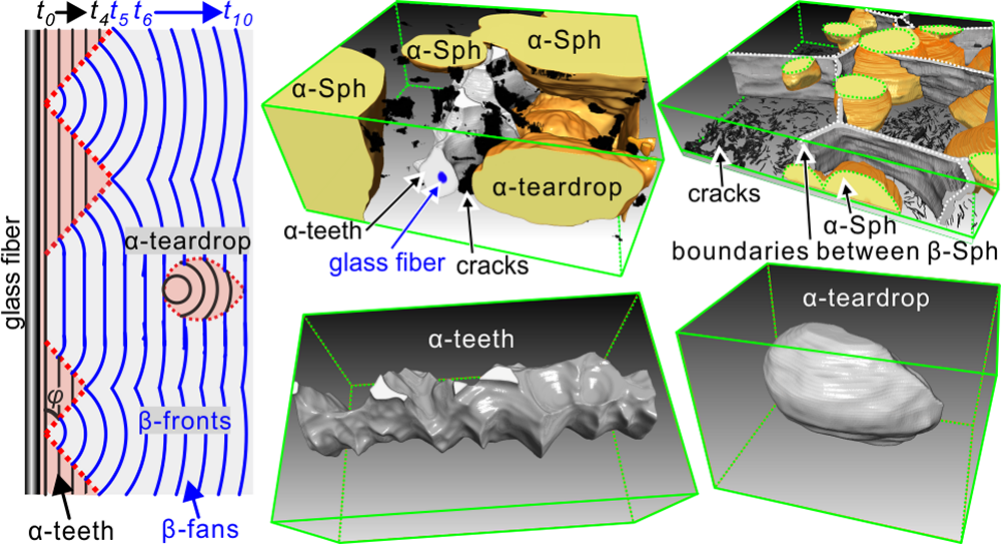

Polymorphism of semicrystalline polymers has significant influence on their physical properties, with each form having its advantages and disadvantages. However, real-life polymer processing often results in different coexisting crystal polymorphs, and it remains a challenge to determine their shape, spatial distribution, and volume fraction. Here, *i-*polypropylene (*i*-PP) sheets containing both α- and β-forms were prepared either by adding β-nucleating agent or by fiber pulling-induced crystallization. By adding a compatible dye that is partially rejected from the growing crystalline aggregates (spherulites and cylindrites), we visualize the shape of these objects in 3D using two-photon fluorescence confocal microscopy. To distinguish between crystal forms, we take advantage of the difference in dye-retaining ability of the α- and β-aggregates. Even in 2D, fluorescence microscopy (FM) distinguishes the two crystal forms better than polarized microscopy. In 3D imaging, the volume fraction and spatial distribution of α- and β-forms in different morphological types could be determined quantitatively. Morphologies described as α-teeth, β-fans, and α-teardrops were visualized for the first time in 3D. Furthermore, internal and surface microcracks were seen to be associated predominantly with the β-form and around the fiber. Spatial distribution of α- and β-forms was also determined by scanning with a synchrotron X-ray beam. Good agreement was obtained with 3D microscopy, but XRD could not match the detail obtainable by the tomography. The work demonstrates the ability of the 3D imaging method to distinguish different crystal forms and their specific morphologies.

## 1 Introduction

Polymorphism in semicrystalline polymers has considerable influence on their physical properties.^1−7^ Isotactic polypropylene (*i-*PP) is a typical example with four common polymorphs: α-, β-, γ-, and δ-forms,^4,8−12^ and a recently reported ε-form.^13^ All these crystal forms contain only one chain conformation, i.e., the threefold helix with a 6.5 Å repeat. The difference in mechanical properties of these polymorphs is caused by the difference in crystal packing of the 3_1_ helices. *i-*PP with predominantly α-form has high elastic modulus,^14^ and the β-form features high impact strength and elongation at break,^15,16^ while the uniquely structured γ-form has high yield stress.^17,18^ Because of these differences, considerable effort has been devoted to tuning polymorphism of *i-*PP to match its intended use. It can be said that conditions for obtaining the different crystal forms are now reasonably well understood. For example, crystallization in a temperature gradient,^19,20^ shear-induced crystallization,^21−23^ and addition of a β-nucleating agent have all been reported to produce the β-form.^24,25^ On the other hand, *i-*PP crystallization under high pressure and random incorporation of a comonomer can result in the γ-form.^4,14,26−28^

In contrast to the achievements in manipulating the polymorphism of *i-*PP, the ability to determine the crystal morphology of the different polymorphs in 3D still remains a significant challenge even if morphology may be at least as important for mechanical properties as crystal form. So far, shape and distribution of different crystal forms have only been observed in 2D projection by using polarized optical microscopy (POM), by surface profiling using atomic force microscopy (AFM), and by transmission and scanning electron microscopies (TEM and SEM) of small sample areas. POM observations, strongly reliant on differences in birefringence, have been widely used in distinguishing α- and β-spherulites. While β-spherulites were found to have higher birefringence, with a tangential slow axis, the less birefringent α-spherulites often have the slow axis radial when crystallized below *T*_c_ = 134 °C.^9,29,30^ However, α-spherulites might change to mixed-type, and their slow axis may change to tangential with increasing *T*_c_.^30,31^ This often makes it impossible to differentiate between α- and β-spherulites. In AFM, TEM, and SEM observations, α-, β-, and γ-form can only be distinguished based on the different arrangements of the lamellae. For example, α-form is known for its mesh-type cross-hatched morphology as the daughter lamellae are set at ∼80° to the chains on the (010) plane of the parent crystal.^30−32^ β-Spherulites contain mainly radial lamellae, while γ-form appears as sporadic long stripes, since the chains are perpendicular to each other.^33^ However, because of the epitaxial relation between β- and α-lamellae,^10,34^ it is not easy to distinguish different crystal forms only based on AFM, TEM, and SEM.

To determine the fraction of different crystal forms in *i*-PP, the most common methods are differential scanning calorimetry (DSC) and wide-angle X-ray scattering (WAXS). DSC relies on different melting temperatures of the different polymorphs. However, in most cases, the melting peaks of the different forms overlap,^24,35^ so that unreliable curve resolution is required. Besides, the β–α and γ–α transitions might occur during the heating scan,^36,37^ causing underestimation of the extent of the metastable crystal forms. WAXS is more reliable for quantitative determination of the fraction of different forms. However, preferred crystal orientation can seriously affect diffraction intensities, jeopardizing the application of this technique.

In order to observe the morphology in 3D, we apply our recently developed optical tomography method based on two-photon confocal microscopy and suitable polymer labeling. The method was developed via an intermediate stage where 2D fluorescence microscopy (FM) was used to visualize spherulites and their growth in *i*-PP, polylactide, and their nanocomposites using the fact that the added fluorescent dye and labeled nanoparticles partially segregate to the edge of the growing spherulites, outlining their shape, inter-spherulite boundaries, and cracks.^38^ The 3D method developed therefrom recently allowed us to obtain 3D images of spherulites^39^ and cylindrites^40^ in polymers and polymer composites for the first time. These studies found a number of unexpected morphologies, such as non-spherical “spherulites,” “bowls,” and “goblets,” as well as elliptical “cylindrites.” While in those studies the polymers existed in only one crystal form, here we investigate whether different crystal forms of *i*-PP could be distinguished by our 3D imaging technique.

In this work, *i*-PP sheets containing both α- and β-forms are prepared by two methods: (i) crystallization from quiescent melt containing β-nucleating agent and (ii) by shear-induced crystallization brought about by pulling a glass fiber through the precursor melt. The former results in a mixture of α- and β-spherulites, and the latter in cylindrites of mixed α- and β-forms around the fiber. The key requirement, that is a difference in fluorescent contrast between α- and β-forms, was met by the fact that the dye initially had a somewhat higher presence in β-spherulites. Thus, in the current study, we succeeded in visualizing different morphologies belonging to different crystal forms and distinguishing between different morphologies belonging to the same crystal form. This includes features such as the 3D “teardrop” shape of an α-spherulite fully embedded inside a β-spherulite, or a serrated α-tooth core around a glass fiber surrounded by an outer β-fan sheath. The selective tomography method also allowed us to determine accurately the volume fraction of each polymorph, unaffected by crystal orientation, but also to distinguish what fraction of that polymorph belongs to what morphology. The current study demonstrates how optical tomography can give new information on the relationship between polymorphism and polymer morphology.

## 2 Results and Discussion

### 2.1 2D Microscopy of β-Nucleated *i*-PP

**Figure 1:**
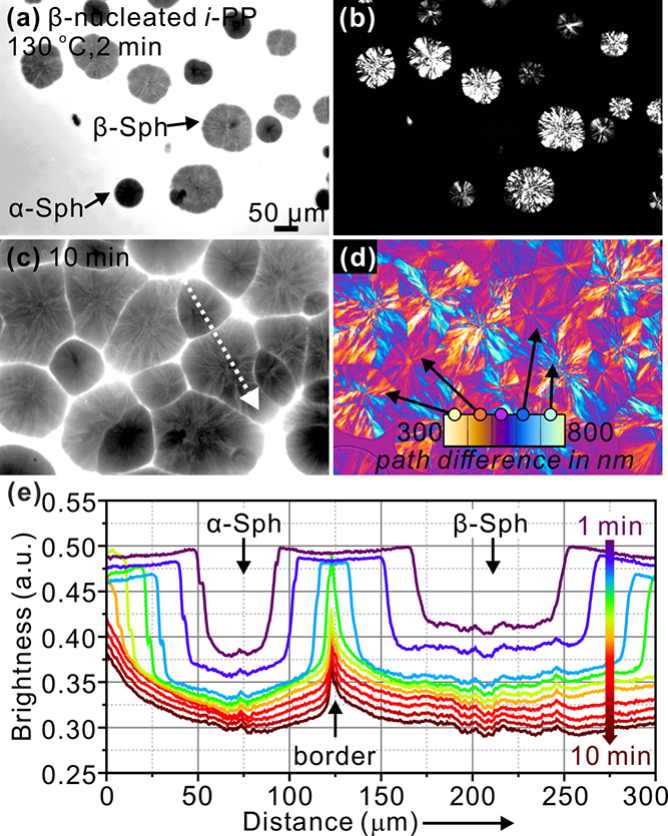
β-Nucleated *i*-PP with 0.05 wt % Nile red crystallized at 130 °C for 5 min (a,b) and 10 min (c,d). (a,c) are FM images, (b) is taken by POM, and (d) by POM with a full-wave (λ) plate. The color scale in (d) shows the path differences between the *e* and *o* ray in nm. (e) Line profiles of fluorescence intensity along the dotted arow in (c). α-Sph and β-Sph denote α- and β-spherulites, respectively.

In general, only α-form can be obtained when *i*-PP crystallizes under normal conditions. To obtain the β-form in *i*-PP, β-nucleating agent was added as this is one of the most effective way to produce β-form. Figure 1a–d shows FM and POM images of β-nucleated *i*-PP crystallized at 130 °C for different times. In FM images (Figure 1a,c), two kinds of dark circles with different contrasts are seen growing within the brighter melt. At the same time, POM images (Figure 1b,d) recorded from the same area as in the FM images also show two types of spherulites, one highly and the other weakly birefringent. Image (d), taken with λ-plate, shows that the former has the slow axis, hence chain axis, tangential to the spherulite, the so-called negative spherulite. The lamellae in those are clearly radial. Meanwhile, the highly birefringent negative spherulites are found to melt at a lower temperature (162 °C, see Figure S1 of SI) than the weakly birefringent ones (175 °C). Combining these facts and the relevant literature,^9,15^ the highly birefringent spherulites can be assigned to the β-spherulites induced by β-nucleating agent, and those with weak birefringence are attributed to α-spherulites. Their low birefringence is due to partial cancellation of path difference coming from radial (dominant) and tangential (subsidiary, or “daughter”) lamellae, characteristic of α-spherulites.^41^ Optical retardation can be estimated from the colors in Figure 1d using the Michel–Levy chart (see inset).

**Figure 2:**
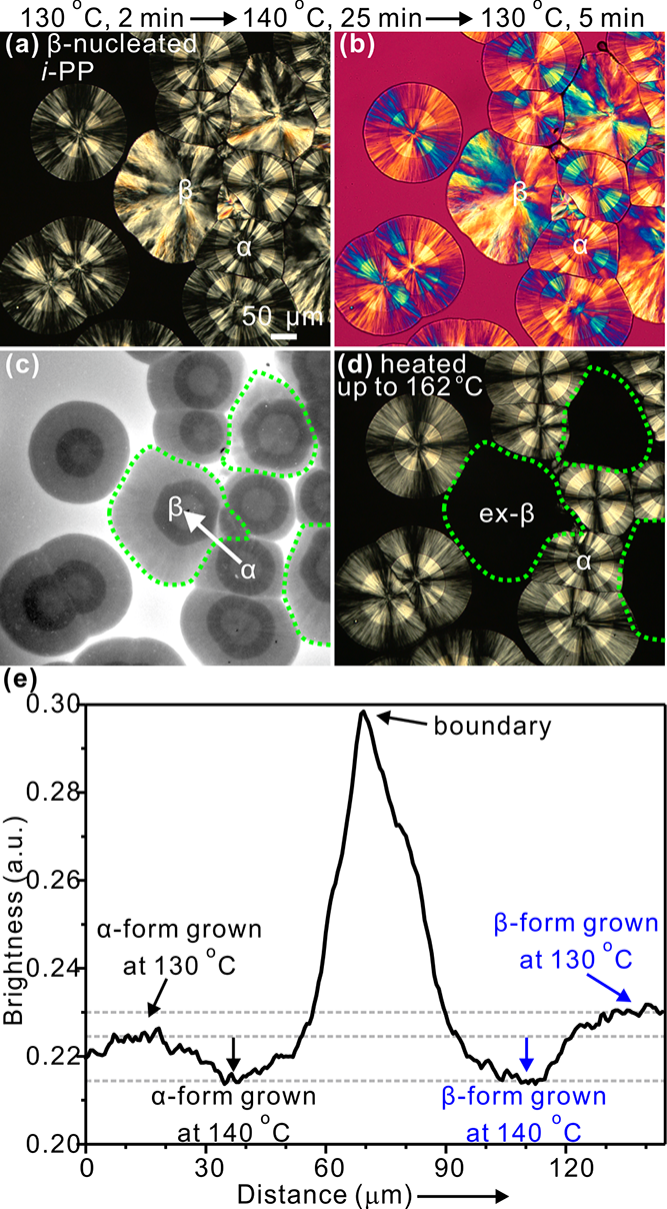
β-Nucleated *i*-PP with 0.05 wt % Nile red crystallized at 130 °C for 2 min, 140 °C for 25 min, and again 130 °C for 5 min in sequence. After crystallization, the sample was heated up to 162 °C to melt the β-spherulites. (a–c) POM without and with a full-wave (λ) plate, FM images, respectively. (d) Sample was heated up to 162 °C. (e) Line profiles of fluorescence intensity along the white arow in (c).

The combination of FM and POM observations makes it apparent that more fluorescence is emitted by β- than by α-spherulites, which is more obvious in Figure 1e where fluorescence intensity profiles are plotted for different times during spherulite growth at 130 °C. As the distinction between the two crystal forms by FM is of prime importance in this work, it would be useful to establish the cause of the FM contrast. While the dye is bound to be rejected from crystals of both forms, there are two possible reasons for the difference in FM contrast: (a) lamellar packing in α-spherulites is denser since the gaps between the dominant radial lamellae are filled by tangential subsidiary lamellae; and (b) β-spherulites grow faster, giving the dye less chance to diffuse away ahead of the spherulite growth front, hence more of it is trapped within the β-spherulite. That β-spherulites grow faster is confirmed in our experiments; at 130 °C, β-spherulites grow ∼47% faster than α-spherulites—see Figure S2. But the difference in growth rate between the two forms diminishes as temperature is increased, to vanish at 140 °C. To answer the above question, we performed the following experiment illustrated in Figure 2.

β-Nucleated *i*-PP was first crystallized at 130 °C for 2 min, then at 140 °C for 25 min, and finally again at 130 °C for 5 min. While visual appearance of β-spherulites is not affected significantly, this temperature regime produced α-spherulites with three distinct circular regions, a weakly birefringent inner and outer ring and a more strongly birefringent middle ring (Figure 2a,b). The stronger birefringence of the middle ring, grown at 140 °C, is in line with the established fact that lamellar branching of α-spherulites is suppressed at high crystallization temperature,^31^ leaving mainly radial lamellae. In the fluorescence image (Figure 2c), those parts of both α- and β-spherulites that were grown at 140 °C are equally dark as also confirmed by the radial intensity scans in Figure 2e. As crystallization temperature was dropped to 130 °C, the β-spherulites became significantly brighter, while α-spherulites showed only a small change. Since β-spherulites maintained essentially the same morphology with radial lamellae at both temperatures, with increased growth rate being the only significant change, we conclude that the main reason for β-spherulites appearing brighter than α-spherulites in FM is the inability of the dye to diffuse fast enough to completely avoid being trapped by the spherulite’s growth front. While growth rate difference between the two spherulite types seems to be the main cause of FM contrast, some effect of lamellar packing density may also play a secondary role. In any event, while FM is very useful in distinguishing α- and β-forms of *i*-PP, it is not applicable for material crystallized at the highest temperature. Incidentally, in Figure 2d, identification of β-spherulites was confirmed unequivocally by their lower melting point.

In addition to being able to identify different crystal forms, FM has a clear advantage over POM in giving an easily recognizable shape of a morphological object of uniform hue, uncomplicated by high-contrast streaks due to fluctuations in birefringence as in POM. Furthermore, because of partial segregation of the dye to spherulite boundaries, boundaries are much more clearly delineated. It can be seen that the boundary lines between β-spherulites are more or less straight (Figure 1c) because all β-spherulites nucleate simultaneously on the nucleating agent and grow at the same rate. However, curved boundaries are seen between α- and β-spherulites, as these grow at different rates, and may have nucleated at different times. Generally, due to their slower growth, α-spherulites are seen to be delimited by convex boundaries. Although 3D shapes of boundaries between spherulites of different forms have been discussed quantitatively in several publications,^29,42−44^ they have never been experimentally observed. They are shown in Sections 2.3 and 2.4.

### 2.2 2D Microscopy Studies of *i*-PP around a Pulled Glass Fiber

**Figure 3:**
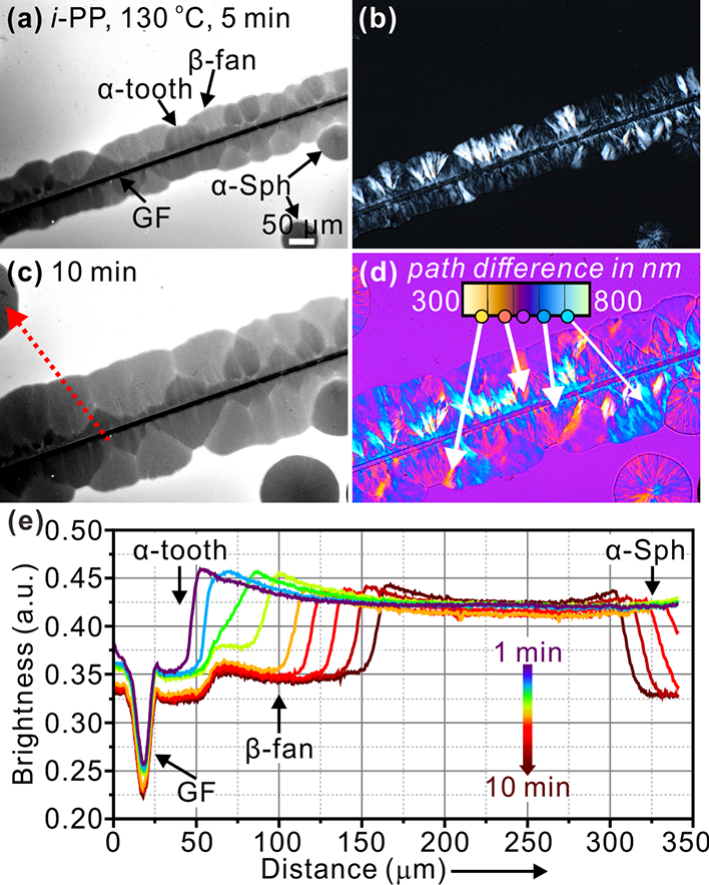
(a–d) Micrographs of *i*-PP with 0.05 wt % Nile red crystallized around a pulled GF at (a,b) 130 °C for 5 min and (c,d) 10 min. (a,c) FM, (b) POM, and (d) POM + λ-plate. The color scale in (d) shows the path differences between the *e* and *o* ray in nm. (e) Line profiles of fluorescence intensity along the dotted red arow in (c).

Flow-induced crystallization is another method to produce β-form in *i*-PP. Here, fiber pull was applied, having the advantage of introducing intense and localized shear flow.^45^ Unlike spherulites formed in β-nucleated *i*-PP, without nucleating agent but with pulling a fiber through the melt prior to cooling induces the formation of cylindrical crystalline aggregates (Figure 3a–d). It should be noted that the glass fiber used in the current work has no nucleating ability for *i*-PP (see Figure S3). Thus, formation of cylindrical objects is entirely due to crystallization induced by fiber shear. The morphological model for fiber pull-induced crystallization in *i*-PP has been proposed in past literature as follows: the high shear rate at the GF-polymer interface induces row nuclei in the form of microfibrillar bundles. The row nuclei were believed to be α-form, as supported by in situ synchrotron X-ray studies.^46^ Then folded-chain lamellae of α- and β-forms were shown to epitaxially grow on the surface of row nuclei, resulting in a cylindrical structure of mixed α- and β-forms.^22,47−49^

In FM images (Figure 3a,c), the cylindrite around the GF is seen to be composed of a darker tooth-like layer and a lighter fan-shaped outer layer. Meanwhile, spherulites with brightness similar to that of the tooth layer are nucleated at a distance away from the glass fiber. Combined with evidence from POM images (Figure 3b,d), the tooth-like layer and the spherulites can be assigned to the low-birefringence α-form. Meanwhile, the fan-shaped layer can be identified as the highly birefringent β-form. Hereafter, these objects will be referred to as “α-tooth” and “β-fan.” The identification as α-tooth and β-fan is further verified by selective melting of the β-form at 162 °C (see Figure S4). The triangular tapered inclusions of α-teeth within the β-fans are attributed to the faster growth of the β-form. For the same reason as for β-nucleated *i*-PP, α-teeth occlude less dye than do β-fans, which is clearly shown by the line profiles of fluorescence intensity (Figure 3e). Again, FM presents clear boundary lines delineating the α-teeth without the need for selective melting as in previous POM observations.^15,23,29^

### 2.3 3D Images of β-Nucleated *i*-PP

**Figure 4:**
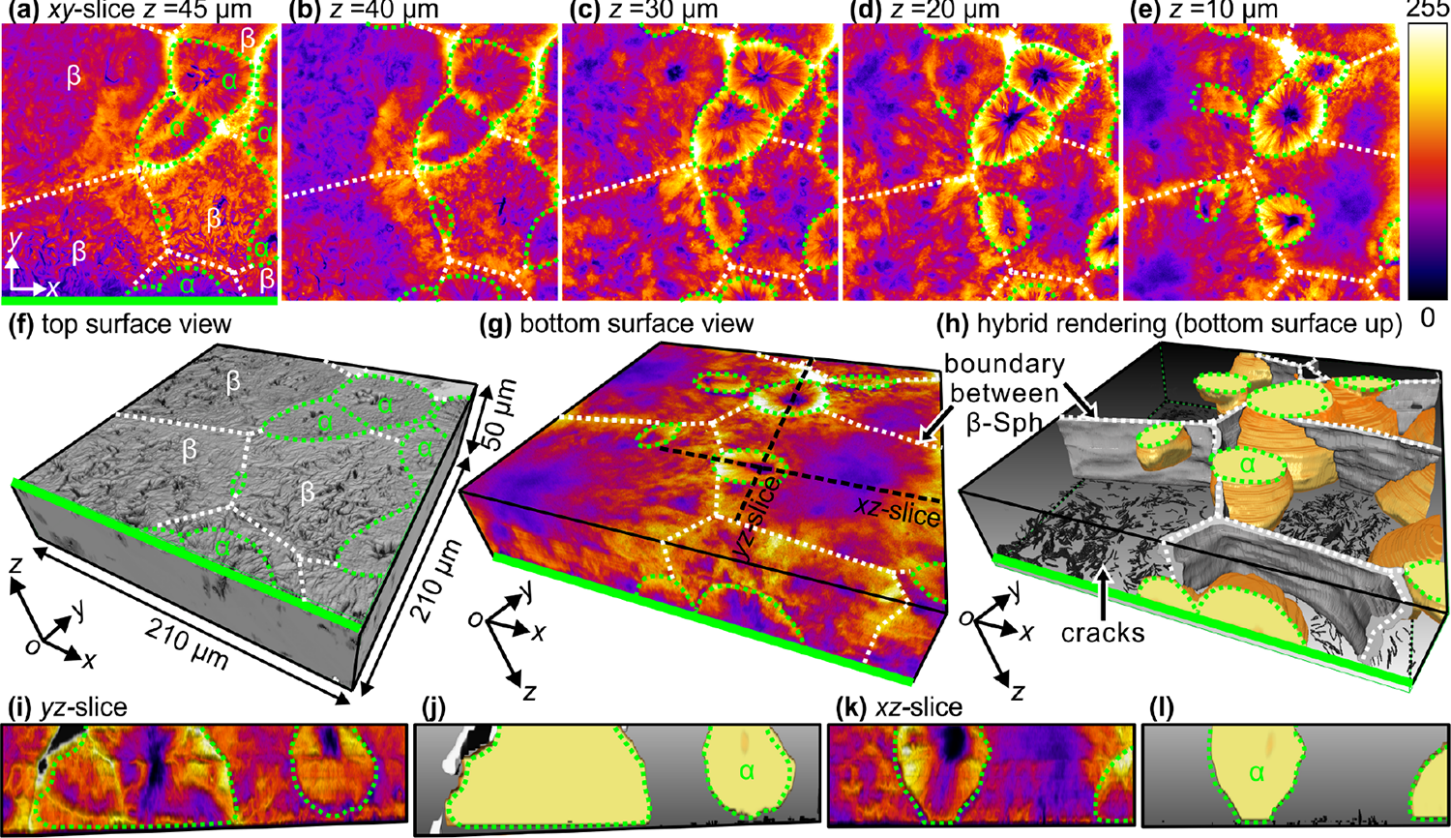
3D images of β-nucleated *i*-PP crystallized at 130 °C for 30 min. (a–e) Representative *xy*-slices from top to bottom. (f) Top surface topography showing the microcracks found predominantly over the entire β-spherulites and over the centers of α-spherulites. (g) Bottom surface view, 3D volume rendering using false-color scale, and (h) 3D hybrid rendering showing the boundary interfaces between β-spherulites (surface rendering, gray), 3D shapes of α-spherulites (orange) and cracks (black) using selected volume rendering. In (a–h), white dotted lines mark β–β boundaries, while green dotted lines mark α boundaries. Note that (g) and (h) are *z*-inverted relative to (f). (i,k) and (j,l) are vertical slices along black dashed lines in (g) from 3D volume and hybrid rendering, respectively.

We now move to 3D imaging using confocal microscopy. Slices parallel to film surface (*xy*-slices) were recorded in 1 μm *z*-increments from the bottom (*z* = 0 μm) to top (*z* = 50 μm) of a β-nucleated *i*-PP sheet. Figure 4a–e shows the representative *xy*-slices using false colors to represent fluorescence intensity. Similar to 2D FM observations, many α-spherulites are leaf-shaped, with convex curved boundaries, fully or partially occluded within β-spherulites. Unexpectedly, the α-spherulites are brighter than β-spherulites, which is opposite of what was seen by 2D FM (Figure 2a,c). The reversal of fluorescence contrast between α- and β-spherulites is interesting and is possibly caused by the dense network of microcracks in β-spherulites near the top surface as shown by the top surface relief (Figure 4f). The dense microcracks scatter the incident laser beam, resulting in relatively low fluorescence emission out of the focal plane in β-spherulites. To verify this conjecture, we performed the following experiment illustrated in the next figure.

In order to get a better view of objects below the cracked surface, the top surface is facing down in the following 3D images. As shown in Figure 4g, β-spherulites occupy most of the volume of the β-nucleated *i*-PP sheet, while α-spherulites are sporadically distributed. In order to show the 3D shapes of α-spherulites inside the film, 3D hybrid rendering (Figure 4h) was applied in which only boundaries between β-spherulites are shown using surface rendering (in gray), while α-spherulites are displayed using volume rendering (in orange). The α-spherulites are located within the β-spherulites or straddle the boundary between them (see Supporting Video 1). The 3D shapes of α-spherulites are rather complicated, showing asymmetrically curved faces. Figure 4i,k and j,l shows vertical slices through an α-spherulite from 3D volume and hybrid rendering, respectively. The asymmetry of curvature of the α-spherulite boundaries is seen in both vertical slices.

The 3D images also allow the volume fraction and distribution of selected morphological features to be quantified. Here, for the β-nucleated polymer crystallized at 130 °C, the volume fractions of α- and β-spherulites, and the microcracks are about 19, 80, and 1%, respectively. Furthermore, we could establish that about 90% microcracks have a volume below 30 μm^3^ (see the percentile chart in Figure S5). Therefore, with the application of 3D imaging, both the cracked surface and the α- and β-spherulites below the surface can be visualized, and their volume fraction determined.

**Figure 5:**
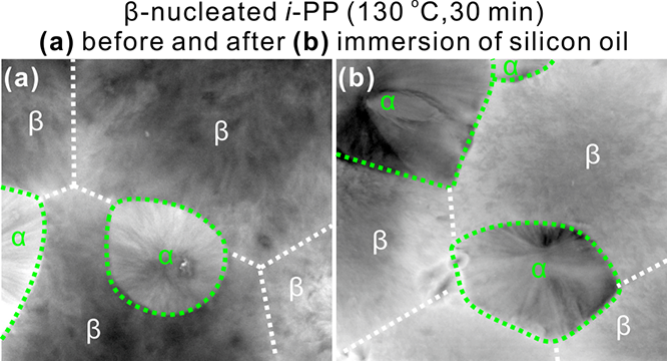
Comparison between two confocal micrographs (*xy*-slice) of the same sample of β-nucleated *i*-PP crystallized at 130 °C for 30 min. (a) Before and (b) after immersion in silicone oil. The weaker fluorescence of β-spherulites in the dry sample (a) is caused by scattering of incident light on their rough surface, full of microcracks. The red FM images were converted to 256 gray levels to improve visual intensity resolution.

The contrast between α- and β-spherulites in 3D imaging (confocal microscopy) is indeed inverted compared to FM. This is because 3D experiments were done after solidification of the samples, while FM observations were made during the growth of α- and β-spherulites. In the former case, cracks had formed during cooling of the fully crystallized material from 130 °C to room temperature. The existence of dense cracks in β-spherulites scatters the incident laser beam, causing fluorescence emission to be relatively low (Figure 5a). To confirm the above proposition, the *i*-PP sample was immersed in silicone oil so as to fill cracks with a medium of similar refractive index as *i*-PP. Αs shown by Figure 5b, after immersion in silicone oil, the scattering of incident laser beam was suppressed and β-spherulites again become brighter than α-spherulites.

### 2.4 3D Images of *i*-PP around a Pulled Glass Fiber

**Figure 6:**
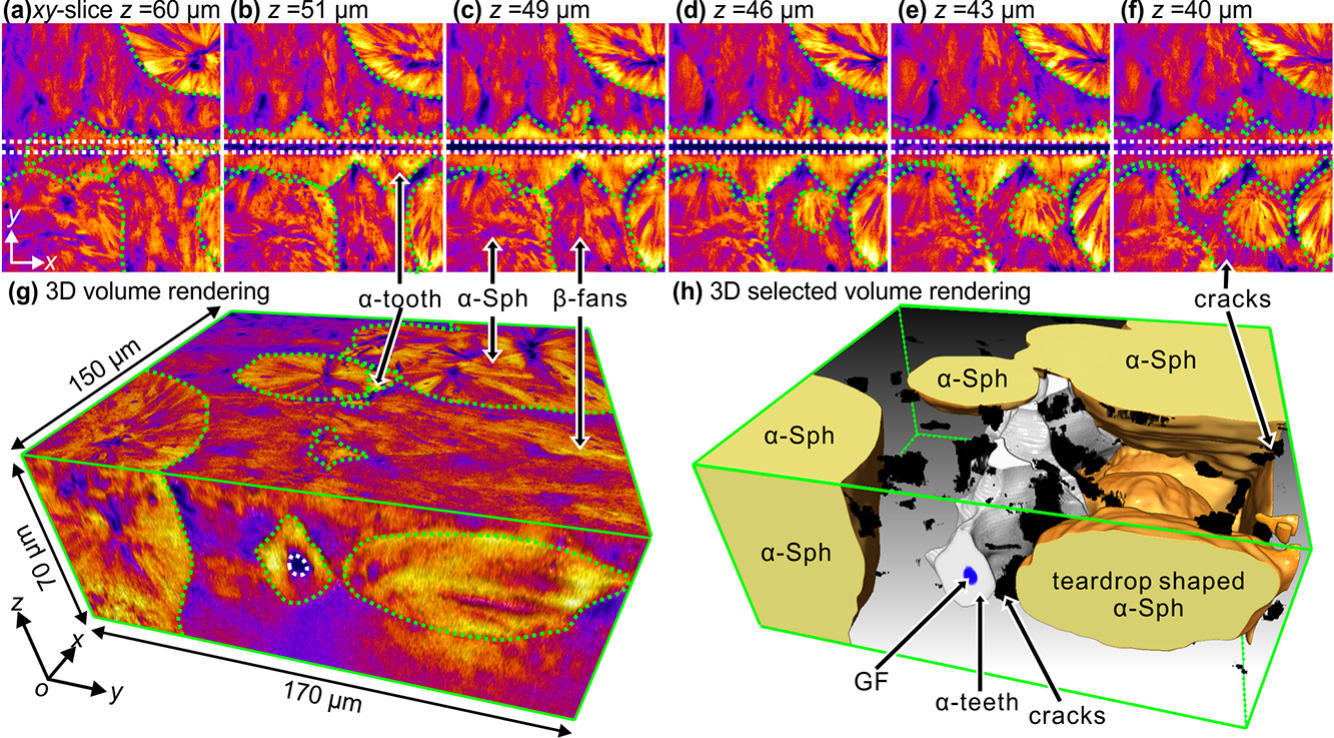
3D images of *i*-PP crystallized for 30 min at 130 °C around a pulled glass fiber. (a–f) Selected *xy*-slices from the top to the bottom layer containing the GF. (g) False-color 3D volume rendition. The boundaries between α-spherulites and β-cylindrites (β-fans), and between α-teeth and the GF are outlined green dotted and white dotted, respectively. (h) Selected volume rendering showing 3D shapes of α-spherulites/teardrop-shaped α-spherulite (orange), α-teeth (gray), the GF (blue), and cracks (black).

3D imaging of *i*-PP cylindrites induced by fiber pull is described next. Figure 6a–f shows the representative *xy*-slices moving from the top to the bottom of the glass fiber embedded in the polymer film. Here we can also see that β-spherulites appear less bright not only compared to α-spherulites but also compared to the α-form parts of the cylindrite as already noted in Figure 4. From the *xy*-slices, different crystalline morphologies can be seen around the pulled glass fiber. The dominant crystalline morphologies are tooth-like α-form objects attached to the fiber and fan-shaped β-form objects making up most of the outer shell of the cylindrite. Further away from the fiber α-spherulites are also seen.

**Figure 7:**
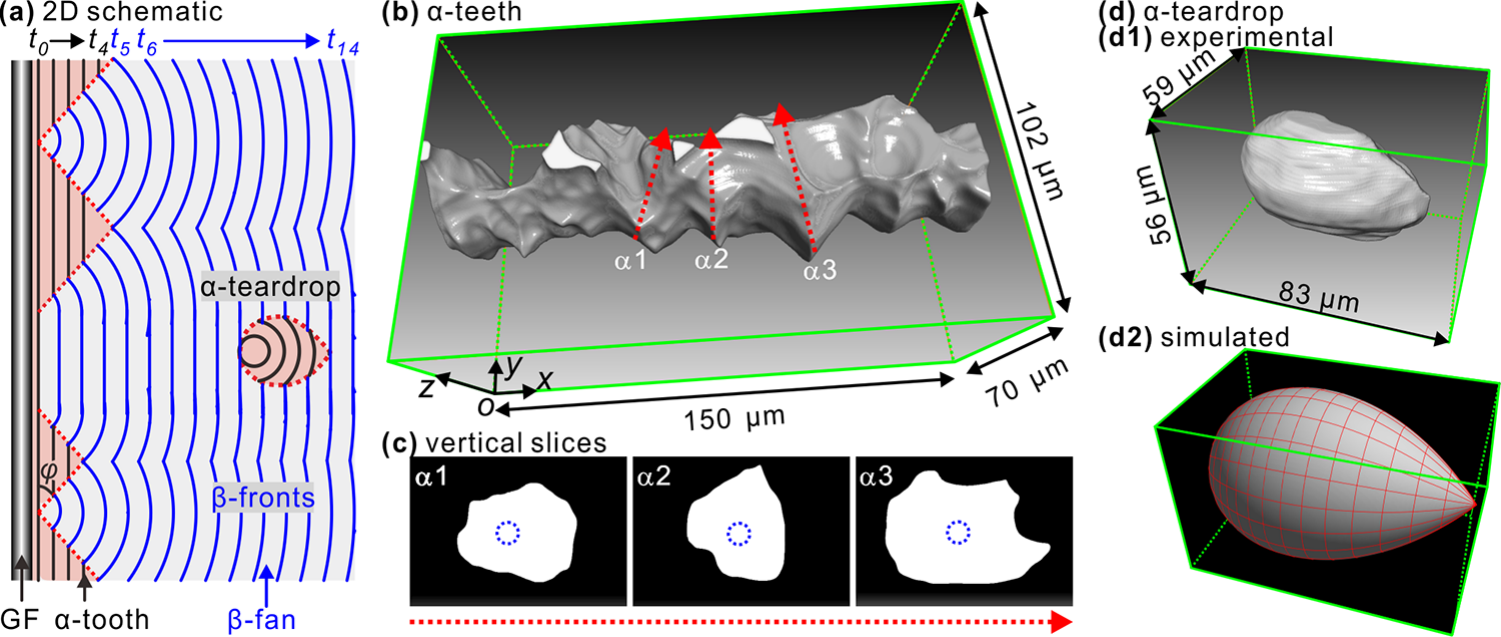
(a) 2D schematic depiction showing the formation of α-teeth, β-fans, and a teardrop-shaped α-spherulite. The increased spacing between isochores reflects the faster growth of β-form. (b) 3D image of α-teeth and (c) vertical slices of α-teeth along dash red lines in (b), the blue dash circles represent the glass fiber. (d) Teardrop-shaped α-spherulite, (d1,d2) experimental and simulation results.

**Figure 8:**
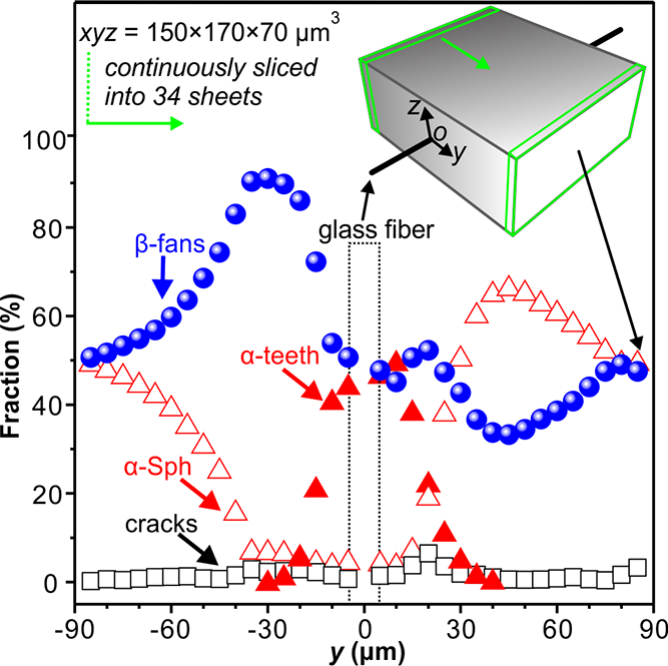
Measured volume fractions of α- (red full triangles) and β-form parts of the cylindrite (blue balloons), as well as of α-spherulites (empty red triangles) and cracks (black squares) plotted as a function of *y*. The glass fiber was located at *y* = 0 μm. Starting from *y* = −85 μm, the fiber pulled *i*-PP sample was divided into 150 × 5 × 70 μm^3^ (*x* × *y* × *z*) slices as exemplified by the green prism in the inset.

Figure 6g shows the false-color 3D volume rendering using the same color palette as in Figure 4a–e,g,i,k. Meanwhile, to make the inside of the sample transparent, selective volume rendering without showing the β-part of the cylindrite is presented in Figure 6h. Clearly, the α-teeth wrap the glass fiber tightly forming the inner layer, which presents a mechanically strong and rough inter-phase between the glass fiber and the matrix. This is believed to be beneficial to mechanical properties of fiber reinforced *i*-PP composites. However, microcracks, occasionally seen at the α-β boundary, may offset this advantage—see below and Figures 6h and 8. Moving further away from the glass fiber, the growth of α-teeth is squeezed out by the occasional but faster growing β-fans. It can also be seen that in some areas, an α-tooth runs directly into an α-spherulite or into the top surface of the film, with no β-fans in between. The 3D shape of α-teeth can be rather complex. This is because the α–β boundary is determined by the growth rate ratio between α- and β-forms and by the distance between the adjacent β-nuclei. Interspersed between α-teeth are β-fans which extend outward until they collide with α-spherulites. Interestingly, several α-spherulites, nucleated ∼19 μm away from the GF surface, become occluded in β-fans, resulting in teardrop-shaped objects,^42−44^ seen here for the first time in 3D (see below and Figure 7d).

Also notable are the large number of microcracks, appearing black in Figure 6h (see also Supporting Video 2). They are observed at the boundaries between α-teeth and β-fans possibly due to their different orientations. The volume of microcracks in the polymer crystallized around the pulled fiber is also larger than in β-nucleated polymer (Figure 4h). The average size of the microcracks is larger, only ∼54% of them with a volume below 30 μm^3^ (∼3 μm linear, see Figure S5). The reason for the difference could be the higher residual stress caused by fiber pulling.^50,51^

Figure 7a shows a 2D schematic drawing of the growing α-teeth, β-fans, and a teardrop-shaped α-spherulite. The black and blue isochores are the growth fronts of α and β forms at increasing times *t*. The ratio between isochore spacings is the experimental ratio of growth velocities *G*_α_/*G*_β_ = (0.14 μm/s)/(0.21 μm/s) = 2/3. The α-teeth are the extension of the “α-shish,” the row-nucleated core immediately surrounding the fiber (*t*_0_). Where there are no interruptions by β-nucleation, the α-phase would have formed a continuous trans-crystalline layer wrapped around the fiber, resulting in an α-cylindrite. However, the favorable molecular orientation in the “shish” gave rise to occasional epitaxial β-nucleation, otherwise very rare on α-spherulites. The β-form growth from a point nucleus results in a straight boundary with the α-cylindrite grown from a line nucleus. As *G*_β_ > *G*_α_, the straight line boundaries of the β-form diverge, hence β-fans. β-Fans eventually squeeze out the α-teeth, leaving them with a triangular shape. The angle (φ) between fiber axis and the straight boundary is given by sin(φ) = (*G*_α_/*G*_β_). Occasionally, β-form growth also starts from a row of nuclei, leaving a bigger gap between α-teeth. Eventually, β-fans join to form an increasingly straight envelope.

While in Figure 7a, the growth of both α-teeth and β-fans starts at *t*_0_, and the α-spherulite, a distance away from the fiber, nucleates later (*t*_8_). At *t*_13_, it is completely occluded by the β-cylindrite with a teardrop shape. Though 2D schematic drawing shows the shape of α-teeth and β-fans, it is still difficult to predict the 3D shape of these objects because the nucleation of β-form on the surface of α-row nuclei is random. Figure 7b shows the 3D image of α-teeth attached to the fiber. It can be seen that the tip of an α-tooth looks like a blade (see Supporting Video 3). The vertical slices across three α-teeth, cut along the red dashed arrows in Figure 7b, also display rounded blade edges with only a few sharp corners (Figure 7c). Figure 7d shows the teardrop-shaped α-spherulite (see Supporting Video 4). It can be seen that the experimental 3D shape in Figure 7d1 is similar to that simulated (Figure 7d2).

From the 3D distribution of fluorescence intensity, one can also calculate the volume fraction and spatial distribution of different crystal forms, different crystal morphologies, and microcracks. As shown by the inset in Figure 8, the volume of the 70 μm thick polymer film was divided into 34 5-μm-thick vertical elementary sheets parallel to the fiber (dimensions *xyz* = 150 × 5 × 70 μm^3^). The fiber was centered at *y* = 0 μm as indicated by the dashed rectangle. Figure 8 shows the *y*-dependence of volume fractions of α-teeth (full red triangles), α-spherulites (empty red triangles), β-fans (blue balloons), and cracks (black squares). The α-tooth volume fraction is maximal (∼45%) in the slice cut through the fiber and decreases steeply when moving away from it (full red triangles). The α-teeth are confined to within 30 μm each side of the fiber. The distribution of β-fan volume is more complex as it was affected by the growth of α-teeth and α-spherulites. β-Fans occupy 49% of the volume within the −5 μm ≤ *y* ≤ 5 μm interval. Moving away from the fiber, the β-fraction (blue balloons) increases to reach a maximum 25–30 μm away from its axis. The increase up to the maximum is a result of the divergent shape of the β-fans, while the decrease thereafter is due to the collision with the surrounding growing α-spherulites. The volume fraction of microcracks is around 1.6% of the total sampled volume, and it peaks 20–30 μm from the fiber.

### 2.5 WAXS Profiling of β-Nucleated *i*-PP

**Figure 9:**
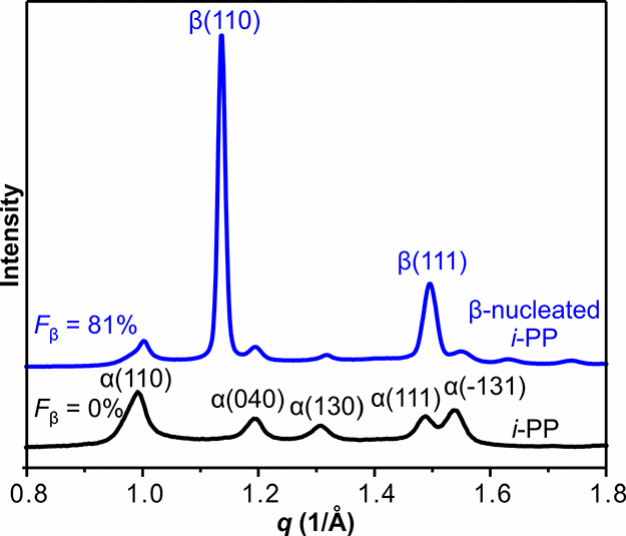
WAXS powder diffractograms of *i*-PP with (blue line) and without β-nucleating agent (black line) crystallized at 130 °C.

The ratio of α- and β-forms in β-nucleated *i*-PP was also measured by WAXS. As shown in Figure 9, β-nucleated *i*-PP displays strong β(110) and β(111) diffraction peaks in powder WAXS, absent in the polymer without nucleating agent. The equation proposed by Turner-Jones et al.^52^ has been widely used to calculate the percentage (*F*_β_) of β-form in nucleated *i*-PP. While the original equation takes into account only β(110) reflection for β-form and α(110), α(040), and α(130) for α-form, neglecting other reflections, here, using the Lorentz-corrected WAXS profile of pure α-form, we have calculated the fraction (*k*_α(110)_) of intensity of the α(110) reflection (*A*_α(110)_) in the total intensity of all Bragg peaks of the α-form ∑*A*_α_ (Figure S6). Then, for *i*-PP with both α- and β-forms, the fraction of β-form can be calculated as:
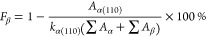
1where *k*_α(110)_ is 0.24, ∑*A*_β_ is the total Bragg intensity of β-form. The *F*_β_ of β-nucleated *i*-PP is ∼81%, in agreement with the 80% volume fraction determined from 3D images.

### 2.6 Wide- and Small-Angle X-ray Scattering (WAXS/SAXS) Experiments on *i*-PP after Fiber Pulling

**Figure 10:**
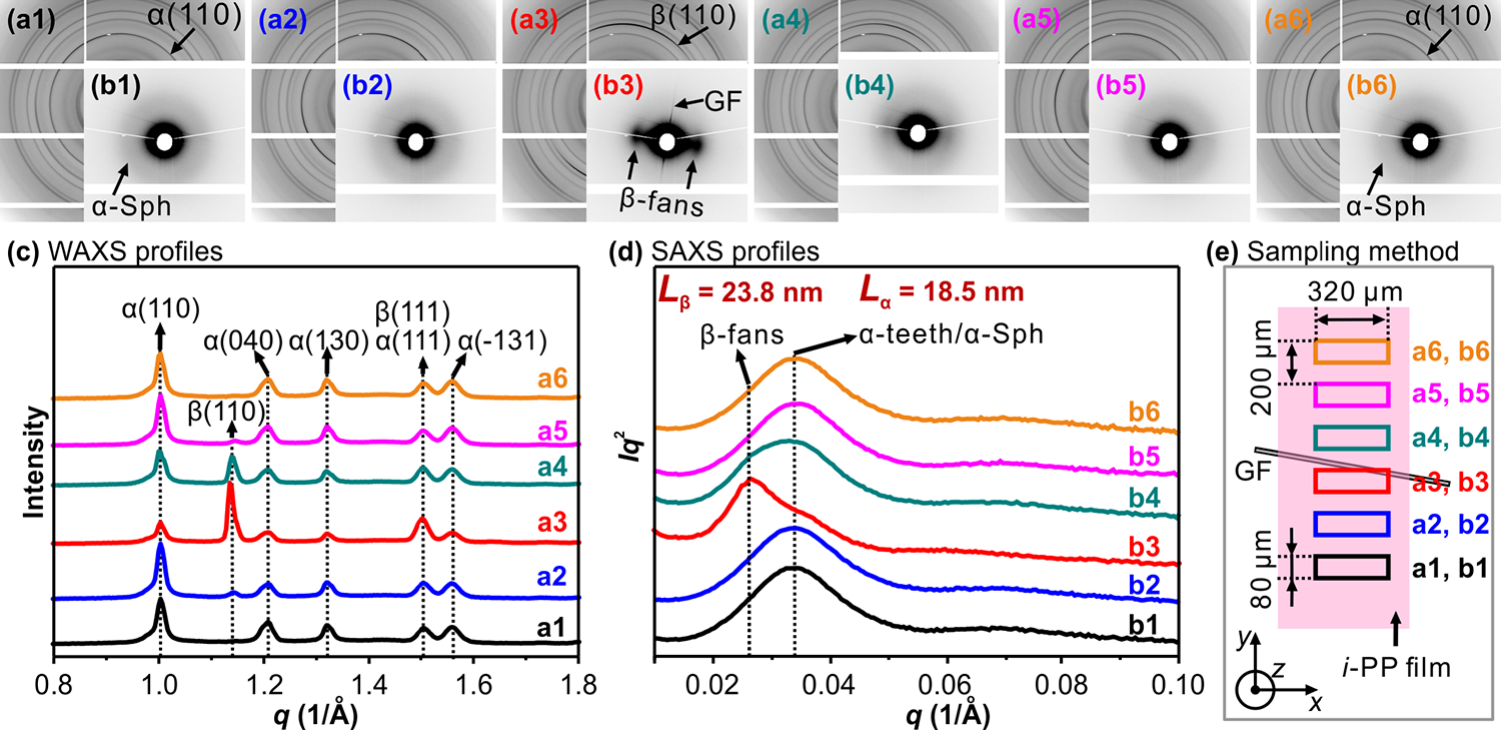
(a1–a6) WAXS and (b1–b6) SAXS patterns of GF reinforced iPP composite crystallized at 130 °C. GF was horizontal as indicated by vertical reflection streaks in (b3). (c) and (d) are the radial scans of the respective WAXS/SAXS patterns. (e) Schematic diagram showing how the X-ray beam scanned across the GF.

Distribution of α- and β-forms across the pulled fiber was measured by scanning a synchrotron X-ray beam of 320 × 80 μm^2^ cross-section perpendicular to the fiber (short beam dimension normal to fiber axis) in steps of 200 μm. Figure 10a1–a6 is the WAXS, and Figure 10b1–b6 is the SAXS patterns recorded at room temperature. Figure 10c,d is the respective WAXS and SAXS intensity profiles. Figure 10e shows the X-ray beam size and scan sequence across the glass fiber. In Figure 10a1,b1, neither WAXS nor SAXS patterns show any preferred orientation, and only diffraction from α-spherulites is seen. As the beam moves toward the fiber (Figure 10a2,b2), both WAXS and SAXS develop anisotropy which culminates at the fiber itself (Figure 10a3,b3). Note that the fiber is tilted by ∼10° from the horizontal direction. WAXS clearly shows the β(110) diffraction arcs in the meridional direction (see the azimuthal distribution of β(110) in Figure S7). The corresponding SAXS pattern (Figure 10b3) displays long period scattering lobes at the equator, indicative of crystalline lamellae normal to the fiber in β-fans. The diffraction from α-form in Figure 10a3 also shows preferred orientation. The fraction of the α(110) reflection intensity around the meridional maximum corresponds to the parent α-crystals (dominant lamellae), also grown radially normal to the glass fiber and the “shish” surrounding it. Non-meridional intensity, attributed mainly to α-daughter lamellae, can be seen in the azimuthal distribution of α(110) in Figure S7. The thin equatorial SAXS streak in Figure 10a3 comes from reflection of grazing X-rays on the glass fiber. Once the X-ray beam crossed the glass fiber, β-form diffraction again gets weaker (Figure 10a4,a5,b4,b5). In Figure 10a6,b6, both WAXS and SAXS lose preferred orientation and WAXS shows only α-form diffractions, coming from α-spherulites. The above WAXS features can also be seen in azimuthally averaged radial intensity profiles in Figure 10c.

Figure 10d shows the SAXS profiles. It can be seen that a diffuse scattering peak at approximately 0.034 Å^–1^ is observed when only α-form exists. The SAXS profile shows a clear additional scattering peak at ∼0.026 Å^–1^ where β-form enters the beam. Therefore, these two scattering peaks can be assigned to the α- and β-forms, respectively. The long periods (*L*) of α and β lamellar stacks are estimated from Bragg equation as 185 and 238 Å. That β-lamellae are ∼30% thicker can be attributed to their lower melting point, hence smaller supercooling of this forms at crystallization temperature.

In the main, X-ray results are consistent with the measurements by 3D imaging. However, even if higher-resolution experiments were done, using a microbeam currently available at synchrotrons, the X-ray method still lacks the third dimension. In principle, X-ray tomography is an option, but to achieve 1 μm, resolution crystallinity of the polymer would be destroyed by radiation damage. One should also bear in mind that the percentage of α- and β-forms obtained by the current X-ray method, especially in the region around the fiber, is inaccurate due to preferred orientation which simple azimuthal intensity integration does not sample correctly. The advantages offered by the current optical tomography are thus manifold, not least in view of the availability of optical confocal microscopes in comparison with that of microbeam facilities at synchrotrons. However, the main advantage of optical tomography in the present context is visualization of detailed 3D morphology of the different crystal forms.

## 3 Conclusions

Unlike our previous optical tomography studies, which involved only one crystal form of a polymer and generally one type of crystalline morphology, in the current work, we have shown that the method can be successfully applied to more complex systems, that is, *i*-PP composed of two different crystal forms and at the same time containing different morphological species (α- and β-spherulites, α-teardrops, α-teeth, and β-fans combined making up a complex cylindrite). The different rendering techniques applied show vividly the 3D shapes, spatial distributions, and volume fraction of α- and β-spherulites in β-nucleated *i*-PP, as well as α and β components of complex cylindrites and α-spherulites in fiber pulled *i*-PP, and also internal and surface microcracks in both. Furthermore, as a single technique, the advantages of crystal form characterization by optical tomography over X-ray measurements were demonstrated in several aspects. Nevertheless, resources permitting, the combination of the two techniques is clearly preferred. We anticipate that the described 3D optical imaging method, with a suitably chosen dye, will be used to obtain previously unavailable information on other polymers and their composites, other morphologies, and polymorphic crystal forms.

## 4 Experimental Section

### 4.1 Materials

*i*-PP (*M*_w_ = 250,000 g/mol and *M*_n_ = 67,000 g/mol), Nile red (NR), and *p*-xylene were purchased from Sigma–Aldrich (USA). A ∼10 μm glass fiber was used without surface modification. The arylamide derivative β-nucleating agent (TMB-5) was provided by the Fine Chemicals Department of the Shanxi Provincial Institute of Chemical Industry.

### 4.2 Sample Preparation

*i*-PP with NR (0.05 wt %) and *i*-PP with NR and β-nucleating agent (0.2 wt %) were prepared by freeze-drying to obtain uniform mixtures, using *p*-xylene as the solvent. Note that the addition of NR does not affect crystallization of *i*-PP as the same type of spherulites with identical growth rates were observed in both neat *i*-PP and *i*-PP with 0.05 wt % NR (Figure S8). Prior to crystallization, β-nucleated *i*-PP samples were melted at 210 °C for 5 min to remove thermal history and then cooled to 130 °C at 20 K/min, followed by isothermal crystallization for 30 min before cooling to room temperature. The experimental procedure for *i*-PP without nucleating agent was similar, except that a glass fiber was inserted between two *i*-PP thin films during melting at 210 °C, which was then pulled at a rate of ∼1 mm/s for 2 s at 145 °C before cooling the sample to 130 °C (Figure S9).

### 4.3 Combined Polarized Optical and Fluorescence (2D) Microscopy

In crystallization experiments, an Olympus BX51 microscope was used, equipped with an Olympus DP74 camera, and a LTS420E heating cell controlled by a T95-HS unit (Linkam). Polarized microscopy was done in transmission with and without a 530 nm λ-plate. Fluorescence micrographs were recorded in reflection using a CoolLED pE-300 White light source, BP 460–490 excitation and LP 520 emission filters, and a DM 500 dichromatic mirror. The color FM images were converted to 256 gray levels to improve visual intensity resolution.

### 4.4 Confocal Microscopy

A Leica SP8 DIVE confocal microscope (Germany) with a Ti-sapphire multiphoton laser was used to record *xy-*slices in 1 μm *z*-steps. The wavelength of the two-photon excitation laser was set at 1000 nm. Three methods were applied to reconstruct the 3D images of crystalline morphologies from the full *z*-stack. These were (i) 3D volume rendering with using false colors to represent fluorescence intensity, (ii) 3D selected volume rendering that only shows the objects within a specified intensity range, and (iii) surface rendering showing surfaces of maximum intensity gradient.

### 4.5 X-ray Measurements

Spatially resolved WAXS/SAXS was recorded at beamline I22 of Diamond Light Source (U.K.).^53^ The sample was scanned vertically across the (horizontal) glass fiber with a 320 × 80 μm^2^ (*H* × *V*) X-ray beam incident perpendicular to the film surface in 200 μm steps. Two Pilatus detectors (Dectris) were used to simultaneously collect WAXS and SAXS. Sample-to-detector distances were 158 and 2201 mm. For β-nucleated *i*-PP sample, only WAXS was recorded using an Anton Paar SAXSpoint 2.0 instrument with an Eiger 2-panel detector. A fraction of the diffraction rings was azimuthally integrated since the instrument only allows recording of a limited section of the WAXS.

### 4.6 Simulation of Teardrop-Shaped α-Spherulite

The 3D shape of a teardrop α-spherulite was simulated based on previous reported 2D equations^44^ with distance between the centers of the two spherulites being 18 μm and growing rates being 0.14 and 0.21 μm/s for α- and β-spherulites, respectively, based on experimental rates. The β-spherulite nucleated 5 s before than α-spherulite. The shape was designed to have rotational symmetry around the line connecting the two centers. The experimental and simulated teardrop shapes in Figure 7d1,d2 are on the same scale.

## Supporting Information Available

Melting of β-nucleated *i*-PP; growth rates of α- and β-forms of *i-*PP; crystallization of *i*-PP around an untouched glass fiber (GF); melting of fiber pull-induced β-fans in *i*-PP; micro-crack volume percentiles of β-nucleated and fiber pulled *i*-PP; ratio coefficient of α(110) reflection in pure α-form; azimuthal distribution of β(110) and α(110) diffraction intensities; comparison of spherulite growth rate between neat *i*-PP and *i*-PP with NR; and temperature program of crystallization experiments (PDF)
3D image of β-nucleated *i*-PP crystallized at 130 °C for 30 min (MP4)
3D image of *i*-PP crystallized for 30 min at 130 °C around a pulled glass fiber (MP4)
3D image of α-teeth (MP4)
3D images of teardrop-shaped α-spherulite (MP4)
