# Supplementary material for: 3D Morphology of Different Crystal Forms in β-Nucleated and Fiber-Sheared Polypropylene: α-Teardrops, α-Teeth, and β-Fans
Source: Macromolecules. 2023 Jul 7;56(14):5502–11. doi: 10.1021/acs.macromol.3c00788 (PMC10373525; doi:10.1021/acs.macromol.3c00788)
Supplement: Supplementary file 1 — ma3c00788_si_001.pdf [file ma3c00788_si_001.pdf]

Supporting Information for

3D Morphology of Different Crystal Forms in β-Nucleated and Fiber-Sheared Polypropylene: α-Teardrops, α-Teeth and β-Fans

Shu-Gui Yang,[†,*] *Liang-Qing Zhang,*[‡] *Changlong Chen,*[†] *Jiaming Cui,*[†] *Xiang-bing Zeng,*[§] *Liying Liu,*[#] *Feng Liu,*[†] *Goran Ungar*[†,§,*]

[†]Shaanxi International Research Center for Soft Matter, State Key Laboratory for Mechanical Behaviour of Materials, Xi'an Jiaotong University, Xi'an 710049, China

[‡]College of Material Science and Engineering, Xi'an University of Science and Technology, Xi'an 710054, China

[§]Department of Materials Science and Engineering, University of Sheffield, Sheffield S1 3JD, UK

[#]Biomedical Experimental Center of Xi'an Jiaotong University Health Science Center, Xi'an 710116, China

S1. Melting of β-Nucleated *i*-PP

The melting process of β-nucleated *i*-PP was observed by polarized optical microscopy (POM). Two types of spherulites were formed in β-nucleated *i*-PP as shown in **Figure S1a**, **1b**. The highly birefringent ones grew faster than the weakly birefringent ones. Furthermore, in the subsequent heating run the highly and weakly birefringent spherulites were melting at 162 °C (**Figure S1c**) and 175 °C (**Figure S1d**), respectively. Combining these facts and the relevant literature,[S1,S2] the highly birefringent spherulites could be identified as β-spherulites induced by β-nucleating agent. Conversely, the weakly birefringent spherulites were identified as α-spherulites.

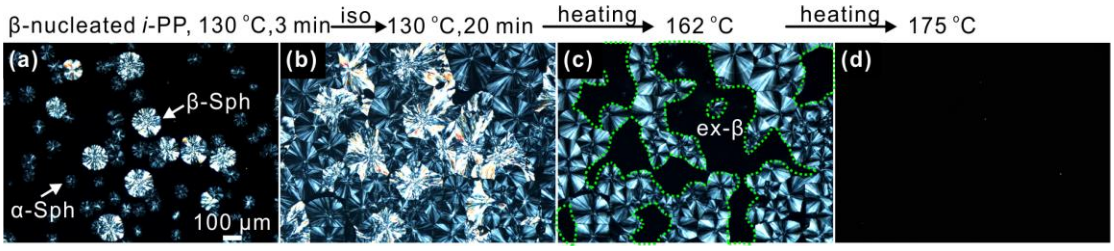

Figure S1. β-Nucleated *i*-PP crystallized at 130 °C for 3 min (a), 20 min (b), then the sample was heated up to 162 °C (c), and 175 °C (d).

S2. Growth rates of α- and β-forms of *i*-PP

Figure S2 shows the growth rates of α- and β-forms. It can be seen that β-form grows faster than α-form over temperature ranges of 130-136 °C. At 140 °C, α- and β-forms grow at a similar growth rate.

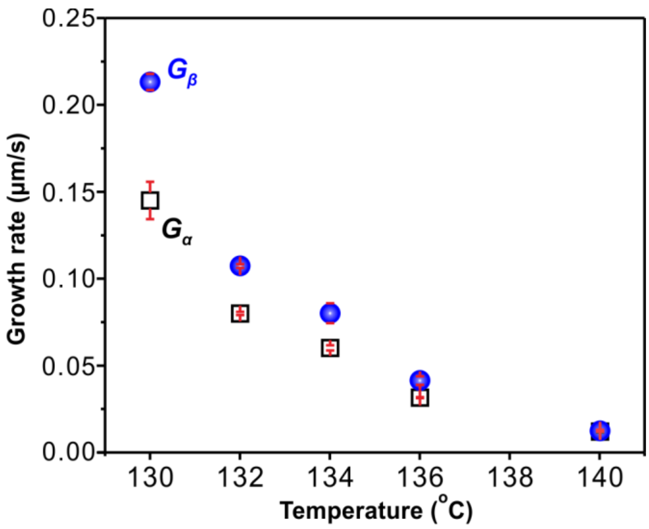

Figure S2. Growth rates of α- and β-forms as a function of temperatures.

S3. Crystallization of *i*-PP around an untouched glass fiber (GF)

POM with a full-wave (λ) plate was used to study the crystallization of *i*-PP around an untouched GF. As shown in **Figure S3**, the spherulites are sporadically distributed in

the field of view. There is no sign of increased nucleation density around the GF, demonstrating that the GF has no nucleating ability for *i*-PP.

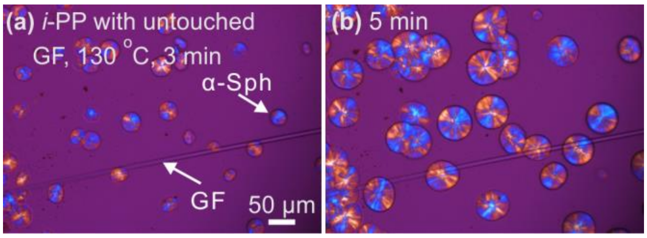

Figure S3. *i*-PP crystallized around an untouched GF.

S4. Melting of fiber pull induced β-fans in *i*-PP

Figure S4 shows the melting of β-fans in *i*-PP around the pulled GF. As the temperature was heated up to 162 °C, the β-fans were melted, and the α-spherulites and α-tooth were preserved.

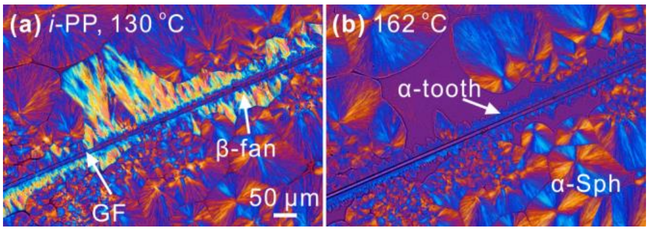

Figure S4. Fiber pull induced cylindrites in *i*-PP heated up to 162 °C after crystallization at 130 °C for 30 min.

S5. Micro-cracks' volume percentiles of β-nucleated and fiber pulled i-PP

Figure S5 compares the percentiles of micro-cracks' volume between β-nucleated *i*-PP and fiber pull in *i*-PP. It can be seen that the micro-cracks in fiber pulled *i*-PP sample have a larger volume than that of β-nucleated *i*-PP. It is about 90% micro-cracks have a small volume below ~ 30 μm^3 for β-nucleated *i*-PP, while for fiber pulled i-PP, only ~ 54% micro-cracks are below ~ 30 μm^3.

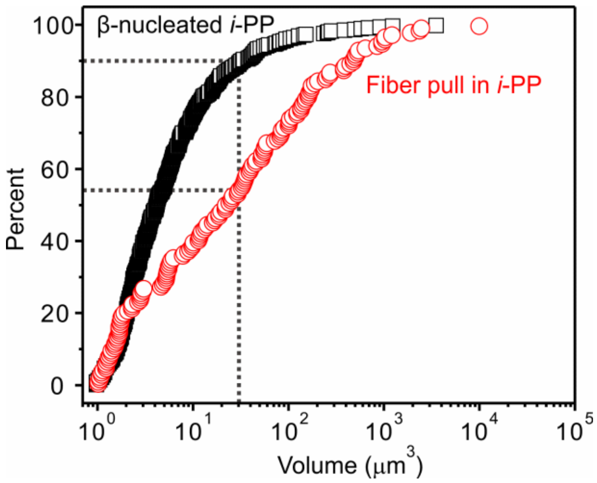

Figure S5. The percentiles of micro-cracks' volume, black square, β-nucleated *i*-PP and red circle, fiber pull in *i*-PP.

S6. Ratio coefficient of α(110) reflection in pure α-form

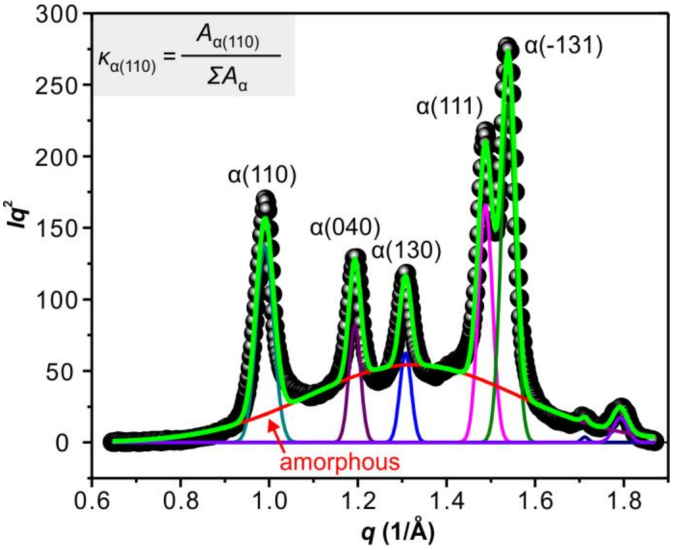

Figure S6. Peak-fitting of the Lorentz-corrected WAXS profile of pure α-form. Inset equation shows how the $k_{\alpha(110)}$ is calculated where $A_{\alpha(110)}$ and $\sum A_{\alpha}$ are the areas of α(110) and the total Bragg peaks of the α-form, respectively.

S7. Azimuthal distribution of β(110) and α(110)

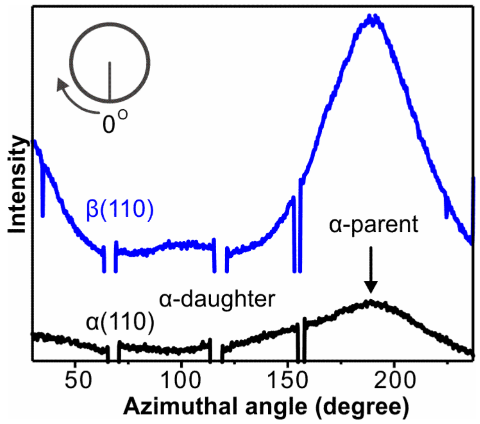

Figure S7. Azimuthal distribution of β(110) and α(110).

S8. Comparison of spherulite growth rate between neat *i*-PP and *i*-PP with NR

The growth of spherulites of both neat *i*-PP and *i*-PP with 0.05 wt% NR were observed by POM. As shown by the insets in **Figure S8**, both neat *i*-PP and *i*-PP with NR were crystallized into low birefringent α-spherulite. Meanwhile, the growth rates of α-spherulite between neat *i*-PP and *i*-PP with NR were identical (within experimental error) at all temperatures (130, 132, 134, 136, and 140 °C). Thus, it can be concluded that the addition of NR does not affect *i*-PP crystallization.

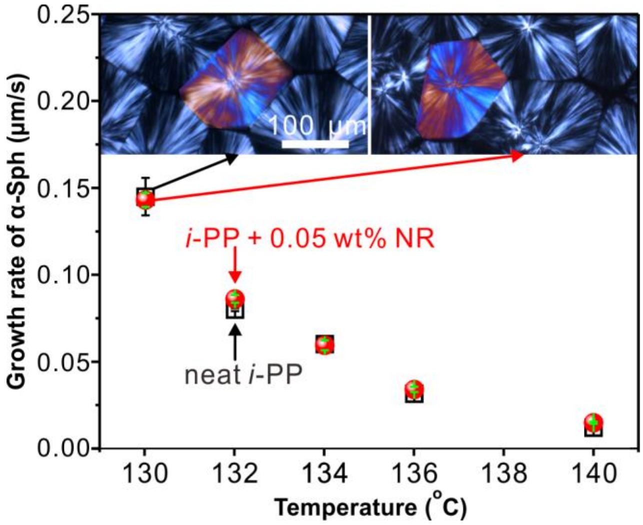

Figure S8. Growth rate of α-spherulites (average of 10 spherulites) as a function of temperature. Insets are representative POM images of neat *i*-PP and *i*-PP with 0.05 wt% NR crystallized at 130 °C for 20 min (crossed polarizers with λ-plate).

S9. Temperature program of the crystallization experiments

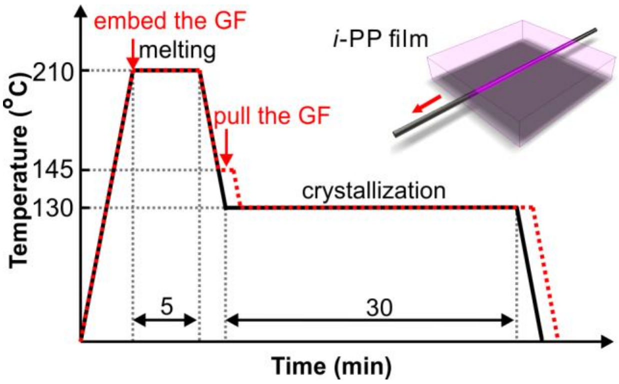

Figure S9. Temperature program of the crystallization experiments. Full black and dotted red lines apply to β-nucleated *i*-PP and fiber pulled *i*-PP samples, respectively.

REFERENCES

[S1] Keith, H. D.; Padden, F. J.; Walter, N. M.; Wyckoff, H. W. Evidence for a Second Crystal Form of Polypropylene. *J. Appl. Phys.* **1959**, *30* (10), 1485-1488.

[S2] Varga, J. β-Modification of Isotactic Polypropylene: Preparation, Structure, Processing, Properties, and Application. *J. Macromol. Sci. Phys.* **2002**, *41*, 1121-1171.
